# Vasculitis in a patient with mevalonate kinase deficiency (MKD): a case report

**DOI:** 10.1186/s12969-021-00645-8

**Published:** 2021-11-22

**Authors:** Ebun Omoyinmi, Dorota Rowczenio, Neil Sebire, Paul A. Brogan, Despina Eleftheriou

**Affiliations:** 1 Infection, Immunity and Inflammation Research and Teaching Department, UCL Great Ormond Street Institute of Child Health, London, UK; 2 National Amyloidosis Centre, UCL Medical School, London, UK; 3 Department of Histopathology, Great Ormond Street Hospital NHS Foundation Trust, London, UK; 4 Paediatric Rheumatology Department, Great Ormond Street Hospital for Children NHS Foundation Trust, London, UK; 5 Centre for Adolescent Rheumatology Versus Arthritis at UCL, London, UK

**Keywords:** Mevalonate kinase deficiency, Autoinflammation, Cutaneous vasculitis, Next-generation sequencing, IL-1 blockade

## Abstract

**Background:**

Mevalonate kinase deficiency (MKD) is a rare autoinflammatory condition caused by biallelic loss-of-function (LOF) mutations in mevalonate kinase (*MVK*) gene encoding the enzyme mevalonate kinase. Patients with MKD display a variety of non-specific clinical manifestations, which can lead to diagnostic delay. We report the case of a child presenting with vasculitis that was found by genetic testing to be caused by MKD, and now add this autoinflammatory disease to the ever-expanding list of causes of monogenic vasculitides.

**Case presentation:**

A 2-year-old male presented with an acute 7-day history of high-grade fever, abdominal pain, diarrhoea, rectal bleeding and extensive purpuric and necrotic lesions, predominantly affecting the lower limbs. He had been suffering from recurrent episodes of fever from early in infancy, associated with maculopapular/petechial rashes triggered by intercurrent infection, and after vaccines. Extensive infection screen was negative. Skin biopsy revealed small vessel vasculitis. Visceral digital subtraction arteriography was normal. With a diagnosis of severe idiopathic cutaneous vasculitis, he was treated with corticosteroids and mycophenolate mofetil. Despite that his acute phase reactants remained elevated, fever persisted and the vasculitic lesions progressed. Next-generation sequencing revealed compound heterozygous mutation in *MVK* c.928G > A (p.V310M) and c.1129G > A (p.V377I) while reduced mevalonate enzyme activity was confirmed suggesting a diagnosis of MKD as a cause of the severe vasculitis. Prompt targeted treatment with IL-1 blockade was initiated preventing escalation to more toxic vasculitis therapies and reducing unnecessary exposure to cytotoxic treatment.

**Conclusions:**

Our report highlights the broad clinical phenotype of MKD that includes severe cutaneous vasculitis and emphasizes the need to consider early genetic screening for young children presenting with vasculitis to exclude a monogenic vasculitis which may be amenable to targeted treatment.

## Background

Mevalonate kinase deficiency (MKD) is an inherited autoinflammatory condition caused by biallelic loss-of-function (LOF) mutations in *mevalonate kinase* (*MVK*) encoding the enzyme mevalonate kinase [1–3]. MKD is now viewed as a phenotypic continuum based on the degree of enzyme deficiency, with mevalonic aciduria (MA) being the most severe phenotype and hyperimmunoglobulinemia D with periodic fever syndrome (HIDS) on the mild end of the spectrum [4]. HIDS is often characterized by lifelong recurrent autoinflammatory episodes, typically lasting anywhere from 3 to 7 days in duration [5]. These episodes are frequently accompanied by various cutaneous manifestations, arthritis, abdominal pain, vomiting, splenomegaly, and lymphadenopathy, with elevated markers of inflammation [1, 5]. Conversely, MA is the more severe phenotype with varying neurological abnormalities such as psychomotor retardation, ataxia, failure to thrive, cataracts, growth retardation and dysmorphic features [6]. The clinical heterogenicity of MKD is therefore well recognised. In addition, patients may often present with a variety of non-specific clinical manifestations mimicking other inflammatory or infectious disorders which can lead to diagnostic delay [7, 8]. We report the case of a child presenting with vasculitis that was found by genetic testing to be caused by MKD thus highlighting the expanding spectrum of phenotypes associated with MKD and now adding this autoinflammatory disease to the ever-expanding list of causes of monogenic vasculitides [9–11].

## Case presentation

**Fig. 1:**
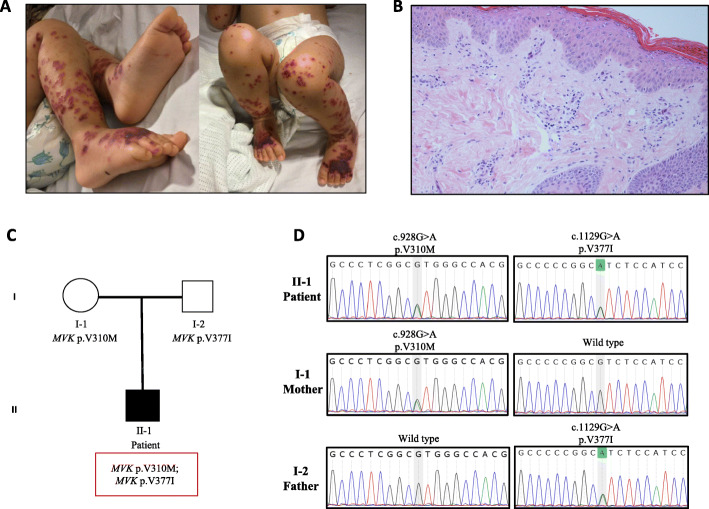
**A.** Palpable purpura mainly affecting the lower limbs in a 2-year-old boy with Mevalonate Kinase Deficiency (MKD). **B.** Skin biopsy of this patient showed vasculitis affecting superficial and deep dermal small vessels in addition to vessels in the subcutis. There is a predominant mononuclear perivascular infiltrate composed of lymphocytes and macrophages, with nuclear dust. (Haematoxylin and Eosin stain, original magnification × 200). **C.** Pedigree of a 2-year-old boy with mevalonate kinase deficiency presenting with vasculitis (black square). Genetic testing revealed compound heterozygosity of c.928G > A (p.V310M) and c.1129G > A (p.V377I) variant in *MVK* gene in this patient. **D.** Sanger sequencing electropherogram of *MVK* c.928G > A (p.V310M) and c.1129G > A (p.V377I) mutations in the patient (II-1) and their heterozygosity in the parents. The c.928G > A mutation was inherited from the mother (I-1), and the c.1129G > A variant was inherited from the father (I-2)

A 2-year-old male of non-consanguineous mixed White/Asian background was referred to us with an acute 7-day history of high-grade fever of up to 40 °C, abdominal pain, diarrhoea, rectal bleeding and extensive purpuric and necrotic lesions, predominantly affecting the lower limbs (Fig. 1A). There was no organomegaly or arthritis noted on clinical examination. There were no concerns about his development and his weight and height were along the 9th centile for his age. Extensive and exhaustive screening for infectious triggers was negative including the following: PCRs for meningococcus, adenovirus, cytomegalovirus, Ebstein-B-virus, mycoplasma, 16S (bacterial), 18S (fungal), Herpes Simplex Virus, parechovirus, parvovirus B19, enterovirus; stool viral screen and NPA respiratory viruses screen; mycoplasma antibodies; anti-streptococcal antibodies; blood cultures and urine cultures; quantiferon test; hepatitis A, B and C screening.

C-reactive protein was grossly elevated (253 mg/L, reference range, RR < 10); erythrocyte sedimentation rate was 41 mm/h (RR < 15); and serum amyloid A was 211 mg/L (RR < 10). Initial full blood count showed: HgB of 92 g/L (RR 105–135 g/L); white cell count of 12.16 × 10 ^9^ /L, (RR 5–15 × 10 ^9^ /L) with neutrophil count of 9.13 × 10 ^9^ /L (RR 1.5–8.5 × 10 ^9^ /L) and platelet count of 288 × 10 ^9^ /L (RR 150–450 × 10 ^9^ /L). Abnormal liver function tests included: alanine aminotransferase of 156 (RR 5–45) U/L and gamma glutamyltransferase of 69 (RR 6–19) U/L; ferritin was mildy elevated at 147 μg/L (RR of < 62 μg/L) while there was normal renal function (urea 4 mmol/L, RR 2.5–6 mmol/L and creatine 15 umol/L, RR < 31 umol/L) and normal clotting screen. Urinalysis showed no proteinuria or haematuria. Autoantibody testing was negative for ANA/ANCA/ENA; levels of C1Q, C3 and C4, immunoglobulins (IgG 6.3 G/L, RR of 3.5–15.70; IgA 1.3, RR of 0.30–1.3 G/L; IgM 0.48, RR of 0.4–2.2 G/L), nitroblue tetrazolium test, and lymphocyte subsets were normal. Chest x-ray was normal (no evidence of lung inflammation), and ultrasonography of abdomen was normal too (specifically no organomegaly and no bowel thickening or other intestinal pathology); echocardiography showed no serositis or coronary artery changes. Computed tomography imaging of his brain was normal, specifically with no evidence of intracerebral calcification. Magnetic resonance imaging and angiography of brain showed no intracerebral pathology or vasculopathy. Ophthalmology review showed no ocular inflammation. Skin biopsy revealed a patchy perivascular infiltrate comprising of lymphocytes, macrophages and neutrophils, nuclear dust and endothelial swelling, but no fibrinoid vasculitis or fibrin thrombi (Fig. 1B**)**. Immunofluorescence was negative for IgG, IgA, IgM, C3 and C1Q. The histology was suggestive of small vessel leucocytoclastic vasculitis, cause undetermined. Visceral digital subtraction arteriography was normal.

With a diagnosis of severe idiopathic cutaneous vasculitis, he was treated with intravenous methylprednisolone (30 mg/kg/day × 3 days) followed by oral prednisolone 2 mg/kg/day, weaning over 8 weeks; and oral mycophenolate mofetil (MMF, 600 mg/m^2^ twice a day). Despite that, his SAA remained elevated at 561 mg/L with CRP of 18 mg/L, fever persisted and the vasculitic lesions progressed.

More detailed past medical history revealed that at aged 3 months old he had a perianal abscess requiring hospital admission and treatment with antibiotics. He also had been suffering from recurrent episodes of fever from early in infancy, associated with maculopapular/petechial rashes lasting 2–6 days every 2 weeks. These episodes were noted to be triggered by intercurrent infection, and after vaccines. In view of this, custom next-generation sequencing using a gene panel for autoinflammation was undertaken (https://www.ucl.ac.uk/amyloidosis/national-amyloidosis-centre/molecular-genetic-testing). This revealed compound heterozygous mutation in *MVK* (NM_000431.2): c.928G > A (p.V310M) and c.1129G > A (p.V377I) (Fig. 1C). Sanger sequencing confirmed the presence of both mutations in the proband and their heterozygosity in the parents (Fig. 1D). Mevalonate kinase enzyme activity measured at the Laboratory Genetics Metabolic Diseases (Amsterdam UMC; https://www.amc.nl), in patient derived lymphocytes was markedly reduced at 5 pmol/ (min.mg protein), reference values (mean ± SD) 213 ± 59 pmol/(min.mg protein), and to the activity of a healthy control sample analysed on the same day 192 pmol/(min.mg protein).

These results confirmed the diagnosis of MKD as the cause of the extensive cutaneous vasculitis. MMF was stopped, and he commenced anakinra (2 mg/kg/day, titrated up to 5 mg/kg/day) with normalisation of his acute phase reactants, and complete resolution of the vasculitis over 3–4 days. Breakthrough periodic fevers are managed with short courses of oral prednisolone (0.5–1 mg/kg/day). Canakinumab therapy was initiated 8 months later with excellent response (no febrile episodes, no cutaneous lesions and normalisation of acute phase response noted at 6 months since treatment initiation).

## Discussion and conclusions

Patients with MKD display a variety of non-specific clinical manifestations, often mimicking other disorders [1, 7]. In particular, leucocytoclastic vasculitis (and other causes of cutaneous vasculitis) have many differential diagnoses in the young, and we now add to this list MKD as a cause of monogenic vasculitis which responds to IL-1 blockade. MKD may also be misdiagnosed as Periodic Fever, Aphthous Stomatitis, Pharyngitis, Adenitis (PFAPA), Behçet’s disease, or inflammatory bowel disease in view of painful oral ulcers, pseudo-pustular skin lesions, gut inflammation and arthritis, delaying targeted treatment with IL-1 blockade [7]. The prompt diagnosis of MKD in this case prevented further escalation of treatment with more toxic vasculitis therapies such as cyclophosphamide, thus reducing unnecessary exposure to cytotoxic treatment. Targeted therapy with IL-1 blockade in this case also facilitated glucocorticoid sparing, minimising toxicity.

It is now increasingly recognised that vasculitis may be a presenting or early-onset feature of an ever expanding list of monogenic autoinflammatory diseases, including: deficiency of adenosine deaminase type 2; STING-associated vasculitis of infancy; and other emerging genetic immunodysregulatory diseases; monogenic defects in complement; and miscellaneous even rarer genetic syndromes [9, 10, 12]. Skin involvement in MKD often manifests with erythematous macules, papules, urticarial lesions, and erythematous nodules [13], but lymphocytic vasculitis with or without immune complexes is also on occasions described mainly in adults with MKD [7]. Cases of severe cutaneous vasculitis such as seen in the case we report herein are however uncommon and may lead to misdiagnosis of MKD as another inflammatory or infectious disorder [11, 12]. For instance IgA vasculitis was considered in the differential diagnosis of the case presented here in view of the cutaneous and gastrointestinal manifestations. The recurrent nature of the fever and vasculitis attacks and persistent acute phase response prompted diagnostic suspicion of an autoinflammatory disorder and subsequently led to a prompt genetic and biochemical confirmation of MKD in this case with immediate therapeutic implications. The use of either anakinra or canakinumab have been found to be efficacious in controlling and preventing flares in most patients with MKD [14, 15] such as for the patient described herein. However, in some cases the disease may not be well controlled and for these patients haematopoetic stem cell transplantation may be a therapeutic option [16, 17]*.* An earlier diagnosis of MKD could have been considered prior to us seeing this patient given the medical history of early onset recurrent fevers associated with maculopapular rashes and some gastrointestinal symptoms, all features of MKD [7, 18].

In summary, our report highlights the broad clinical phenotype of MKD that includes severe cutaneous vasculitis and emphasizes the need to consider early genetic screening for young children presenting with vasculitis to exclude a monogenic vasculitis which may be amenable to targeted treatment.

## Data Availability

All data generated or analysed during this study are included in this article.

## Abbreviations

MKD: Mevalonate kinase deficiency
MVK: Mevalonate kinase
HIDS: Hyperimmunoglobulinemia D with periodic fever syndrome
PFAPA: Periodic Fever, Aphthous Stomatitis, Pharyngitis, Adenitis
CRP: C-reactive protein
SAA: Serum amyloid A
RR: Reference range
MMF: mycophenolate mofetil
